# 

**Published:** 1877-03

**Authors:** 


### Contents of March Number.

### ORIGINAL.

ART. I.—Puerperal Convulsions. Cases with Symptoms and Treatment. By ———— Fordyce, ........ 163
ART. II.—Abstract of Proceedings of Buffalo Medical Association, Jan. 2d, 1877........ 169

### MISCELLANEOUS.

Croup and Diphtheria........ 172
Compulsory Medication of Prostitutes by the State........ 177
Macro-Microscopical Sections of the Brain........ 178
Excision of Rectum for Carcinoma. Dr. R. J. Levis, Penn Hosp., Phil. 179
The first Telegraph........ 180
A New Poison,........ 181
The Indications afforded by the Pupil,........ 182
Salicylic Acid in Acute Rheumatism,........ 184
Public Health Questions in the United States—Extract from Address. By J. M. Toner, M. D.,........ 185
Sending Plants to Sleep,........ 190
New Method of Treating Urethral Strictures,........ 190
Advanced Thinkers,........ 191
Antizymotic Treatment of Diphtheria,........ 191
A Bloodless Operation,........ 192
Therapeutic Action of Carbolyzed Camphor,........ 192

### EDITORIAL.

Buffalo Medical and Surgical Journal,........ 193
Annual Commencement of the Buffalo Medical College,........ 194
Third Annual Meeting of the Alumni Association of the Medical Department of the University of Buffalo,........ 195
United States Pharmacopœia,........ 197
Bogus Diplomas,........ 180
Back Numbers,........ 198
Books Reviewed,........ 198
Books and Pamphlets Received,........ 202

THE BUFFALO

**Medical and Surgical Journal**

